# Probabilistic Model of Transition between Categories of Glucose Profiles in Patients with Type 1 Diabetes Using a Compositional Data Analysis Approach †

**DOI:** 10.3390/s21113593

**Published:** 2021-05-21

**Authors:** Lyvia Biagi, Arthur Bertachi, Marga Giménez, Ignacio Conget, Jorge Bondia, Josep Antoni Martín-Fernández, Josep Vehí

**Affiliations:** 1 Campus Guarapuava, Federal University of Technology—Paraná (UTFPR), 85053-525 Guarapuava, Brazil; lyviar@utfpr.edu.br (L.B.); abertachi@utfpr.edu.br (A.B.); 2 Diabetes Unit, Endocrinology and Nutrition Department Hospital Clínic de Barcelona, 08036 Barcelona, Spain; gimenez@clinic.cat (M.G.); iconget@clinic.cat (I.C.); 3 Centro de Investigación Biomédica en Red de Diabetes y Enfermedades Metabólicas Asociadas (CIBERDEM), 28029 Madrid, Spain; 4 Instituto Universitario de Automática e Informática Industrial, Universitat Politècnica de València, 46022 València, Spain; jbondia@isa.upv.es; 5 Departament d’Informàtica, Matemàtica Aplicada i Estadística, Universitat de Girona, 17003 Girona, Spain; josepantoni.martin@udg.edu; 6 Institut d’informática i Aplicacions, Universitat de Girona, 17003 Girona, Spain

**Keywords:** type 1 diabetes, compositional data analysis, decision support system, diabetes management, probabilistic model of transition

## Abstract

The time spent in glucose ranges is a common metric in type 1 diabetes (T1D). As the time in one day is finite and limited, Compositional Data (CoDa) analysis is appropriate to deal with times spent in different glucose ranges in one day. This work proposes a CoDa approach applied to glucose profiles obtained from six T1D patients using continuous glucose monitor (CGM). Glucose profiles of 24-h and 6-h duration were categorized according to the relative interpretation of time spent in different glucose ranges, with the objective of presenting a probabilistic model of prediction of category of the next 6-h period based on the category of the previous 24-h period. A discriminant model for determining the category of the 24-h periods was obtained, achieving an average above 94% of correct classification. A probabilistic model of transition between the category of the past 24-h of glucose to the category of the future 6-h period was obtained. Results show that the approach based on CoDa is suitable for the categorization of glucose profiles giving rise to a new analysis tool. This tool could be very helpful for patients, to anticipate the occurrence of potential adverse events or undesirable variability and for physicians to assess patients’ outcomes and then tailor their therapies.

## 1. Introduction

Type 1 diabetes (T1D) is an autoimmune disease characterized by the destruction of pancreatic beta cells. Individuals with T1D rely on external insulin to regulate blood glucose (BG) levels. Insulin infusion can be performed either with multiple daily injections (MDI) or with continuous subcutaneous insulin infusion (CSII). To avoid both high and low levels of BG (hyper- and hypoglycemia, respectively), insulin must be properly infused. Both hyper- and hypoglycemia lead to several complications over time: blindness, kidney failure, cardiovascular complications, and even death [1].

Insulin dosing is usually adjusted by physicians according to patient’s characteristics, such as carbohydrate intake and body weight [2]. Even though current T1D technology allows the combination of continuous glucose monitoring (CGM) and CSII, achieving optimal glycemic control is very complicated due to large intra-patient variability [3]. Changes in glycemia are consequences of the circadian rhythm of hormones responsible for the glucose metabolism and carbohydrate intake, and glycemic variability is increased in people with diabetes [4]. Dealing with the complex behavioral characteristics of patients with T1D makes it difficult for physicians to adjust proper insulin dosing profiles to handle patient’s activities. The effective integration of relevant clinical data in a decision support system (DSS) would be able to relieve the burden that affects physicians in taking clinical decisions during consultations of patients with T1D and would help optimize insulin delivery therapy [5]. The automatic adaptation of insulin therapy based on information from continuous glucose monitoring has been the subject of many developments in the field of DSSs and the artificial pancreas [6,7].

The time spent in, above and below the target glucose range are commonly presented for descriptive purposes and to encourage patients with T1D to increase the time spent in their target ranges, which would improve the quality of glucose control. According to Tyler and Jacobs (2020), increasing from 50% to 60% the amount of time in a day that a person’s glucose is within the target glucose range would be considered to be impressive performance for a DSS [7]. The time spent in different glucose ranges indicate the occurrence of different levels of hypo- and hyperglycemic events, and these times, during a day, are relative contributions to the 24-h time budget. Several works presented approaches for different activities performed during a 24-h period using Compositional Data (CoDa) analysis [8,9,10,11,12,13,14,15]. Since times spent in different glucose ranges are codependent and carry only relative information, log-ratio techniques for CoDa are appropriate to deal with this type of data.

Recently, a methodology based on CoDa analysis for the categorization of daily glucose profiles patients with T1D has been presented in [13]. Glucose profiles of 6-h duration have also been categorized using CoDa [15]. In this work, we aim to present a methodology for the categorization of 24-h and 6-h periods of glucose data, also based on a CoDa approach. It is an improved and extended version of the works presented in [13,14,15]. Moreover, this work also proposes a discriminant model able to predict the category of the last 24-h glucose composition at different times of the day. In addition, it is introduced a probabilistic model of transition between categories from the previous 24-h period to the subsequent 6-h period, based on the retrospective analysis of the glucose data. The idea is to introduce a new tool that would allow patients to know the category of their current glucose profile, and that could be used to help physicians to provide individualized adjustments in patients’ therapies according to each possible scenario of transition between categories of profiles. This work is organized as follows: Section 2 briefly explains some concepts of CoDa. Section 3 summarizes the dataset used and the methodology considered for the analysis of glucose profiles and obtainment of the probabilistic model. In Section 4 the results are discussed. Finally, in Section 5, conclusions are provided.

## 2. Compositional Data Analysis

A compositional vector x of D parts is described as:

(1) $$x=\left[x_{1},\ldots ,x_{D}\right]$$

where $x_{1},\ldots ,x_{D}$ are positive components and $\sum_{i=1}^{D}x_{i}=C$, where *C* is a non-informative closure constant. The simplex ($S^{D}$) is the set of real positive vectors closed to a constant. Unconstrained multivariate data should be analyzed through standard multivariate analysis, which is not valid for compositional data [16]. It was only in 1982 that John Aitchison [17] proposed a methodology for the analysis of compositional data, which considers that the relevant information of a composition consists of the ratios between its components.

The absence of satisfactory parametric classes of distributions and the lack of meaningful definitions of independence for sets of proportions makes it difficult to handle statistics in the simplex [17]. Compositional data in the simplex can be transferred to the real space through the expression in coordinates, on which traditional statistical methods can be applied [18]. These coordinates can be obtained through the *centered log-ratio* (clr) transformation and *isometric log-ratio* (ilr) transformation:

(2) $$\mathrm{clr}\left(x\right)=\left[\ln\left(\frac{x_{1}}{g(x)}\right),\ldots ,\ln\left(\frac{x_{D}}{g(x)}\right)\right]$$

where g(x)=∏j=1…Dxj11DD is the geometric mean of **x**. This transformation was introduced by [18] and projects $S^{D}$ to the real space $R^{D}$ The ilr vector can be viewed as the coordinates of a composition with respect to an orthonormal basis on the simplex [19].

(3) $$\mathrm{ilr}\left(x\right)=\left[\langle x,e_{1}\rangle ,\ldots ,\langle x,e_{D-1}\rangle \right]$$

where $e_{1},\ldots ,e_{D-1}$ is an orthonormal basis in $S^{D}$.

The Aitchison distance [20] between compositions is equal to the Euclidean distance between coordinates, which allows the usage of distance-based clustering techniques in compositional data [21].

## 3. Materials and Methods

### 3.1. Dataset

Data from six T1D patients who wore for approximately eight weeks the Paradigm Veo system with the second generation of the Enlite CGM sensor (Medtronic Minimed, Northridge, CA, USA) were analyzed in this work. Data were collected during a clinical trial that aimed to assess an artificial pancreas system during aerobic and anaerobic physical activity [22]. Demographic characteristics are shown in Table 1. All patients provided written informed consent for research participation.

Patients used a CGM system which recorded BG measurements every five minutes. Periods of different duration were selected for analysis at different times of the day. Glucose time-series of 24-h and 6-h duration starting at different times (00:00, 06:00, 12:00, and 18:00) were analyzed considering the standardized clinical levels of hypo- and hyperglycemia presented in Table 2.

The four levels of hypo- and hyperglycemia presented on Table 2 and the range related to normoglycemia (70 mg/dL ≤ BG ≤ 180 mg/dL) defined the five glucose ranges considered for the analysis of time-series, which allowed the obtainment of the composition:

(4) $$x=\left(G.54,G.54.70,G.70.180,G.180.250,G.250\right)$$

Both 6-h and 24-h periods of glucose data were split into time spent in the five preceding glucose ranges. However, either periods of 6-h or 24-h sometimes presented a non-uniform number of samples, due to missing values related to CGM malfunction. For a 24-h period to be considered valid, each one of its four 6-h periods must contain at least 70% of valid data. In the case of profiles with missing CGM samples, the amounts of time corresponding to the number of missing samples were assumed to be evenly distributed between the existing ranges of the period in analysis. Time spent in different ranges during a finite period of 24-h or 6-h are relative contributions to glucose profiles, codependent, and therefore, should be analyzed as CoDa.

Initially, 24-h periods of glucose data, starting at 00:00 and ending at 24:00 were analyzed, following an adaptation of the methodologies presented in [13,14]. Table 3 reports the average amounts of time spent in each glucose range per patient in minutes/period, considering the profiles from 00:00 to 24:00. These values were obtained by adjusting the geometric means of the components to 1440 min (24-h period). Due to the difference in patients’ central tendency of the compositions of days from 00:00 to 24:00, shown in Table 3, it was performed an individualized analysis of each patient’s profiles.

After the analysis of 24-h periods from 00:00 to 24:00, 24-h glucose time-series starting at 06:00, 12:00, and 18:00 were also analyzed, following the same missing data exclusion criteria as aforementioned. Table 4 shows the quantity of 24-h periods followed by a 6-h period at different times for each patient.

### 3.2. Data Analysis

**Figure 1:**
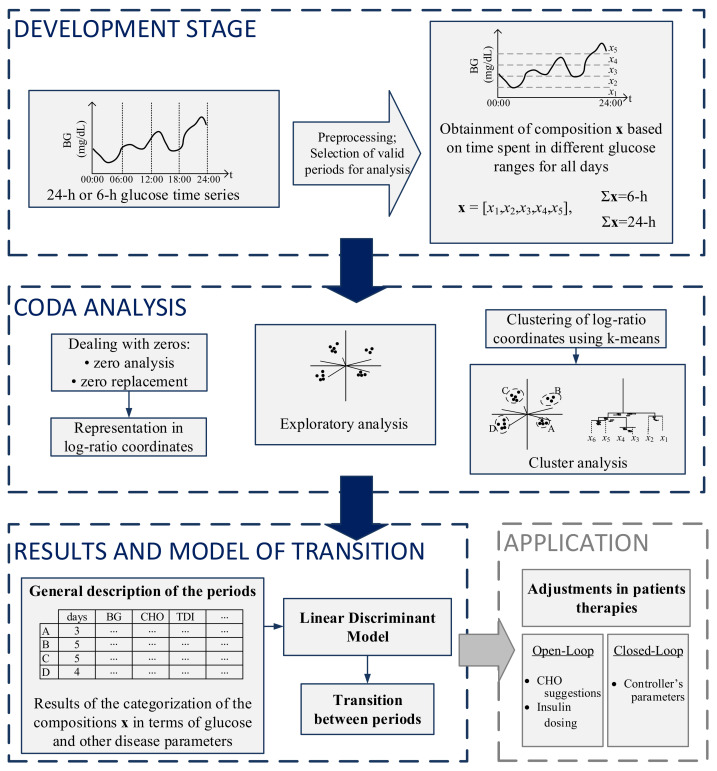
Summarized methodology for the analysis and categorization of glucose profiles using CoDa.

The steps for the analysis and categorization of both 24-h and 6-h periods are presented in Figure 1. The first part of the figure shows the development stage, in which preprocessing and selection of valid days for analysis occur. The second part consists of the zero analysis, followed by CoDa analysis and clustering. The third part consists of the presentation of results obtained in the CoDa analysis in terms of clinical outcomes, the discriminant model to categorize the previous 24-h period at different times of the day, and a transition model to predict the future 6-h period. On the right bottom, a potential application of the methodology is presented.

After the obtainment of the compositions, zero analysis was performed. Since CoDa analysis is based on logarithms of ratios and both operations require non-zero elements in the data matrix, the log-ratio methodology must be preceded by proper handling of zero values, as has been extensively described by [25,26,27,28]. The zero patterns were replaced using the log-ratio Expectation-Maximization algorithm [29]. Given that the CGM records data every 5 min, the matrix of detection limits used in the zero imputation was obtained considering fractions of 5 min, depending on the position of the zero on the compositions, according to [13].

Following the zero replacement, data were represented through coordinates, according to Equations (2) and (3). The orthonormal basis e of the ilr-transformation was defined following a sequential binary partition (SBP) [30], presented in Table 5. The SBP was defined following the clinical interpretation of time spent in different glucose ranges which represent different situations (occurrence of hypo- and hyperglycemic events), as has been detailed in [13].

The first coordinate (*ilr*_1_) is calculated following the expression:

(5) ilr1=65lnG.54·G.54.701122G.70.180·G.180.250·G.2501133

The *ilr*_1_ can be interpreted as a balance between the log-ratio of the geometric mean of the times spent in <54 and 54–70 mg/dL (with +1 at the first line in the sign matrix of Table 5) and the geometric mean of all the other times (with −1 in the sign matrix). It can be interpreted as the relationship between the time spent in the hypoglycemic ranges and the time spent in the normo- and hyperglycemic ranges.

The second coordinate (*ilr*_2_, corresponding to the second row of Table 5):

(6) $${\mathrm{ilr}}_{2}=\sqrt{\frac{1}{2}}\ln\frac{G.54}{G.54.70}$$

The *ilr*_2_ is equal to the log-ratio of the times spent in <54 and between 54–70 mg/dL and can be interpreted as the balance between the time spent in level 2 and level 1 hypoglyemia.

The *ilr*_3_ is the balance between the log-ratio of the geometric mean of the times spent between 180–250 and >250 mg/dL and the geometric mean of the times spent between 70–180 mg/dL, i.e., the balance between time spent in the hyperglycemic and normoglycemic ranges:

(7) ilr3=23lnG.180.250·G.2501122G.70.180

The *ilr*_4_ is equal to the log-ratio of the times spent in >250 and between 180–250 mg/dL. It is the ratio between the time spent in level 2 and level 1 hyperglycemia:

(8) $${\mathrm{ilr}}_{4}=\sqrt{\frac{1}{2}}\ln\frac{G.250}{G.180.250}$$

**Figure 2:**
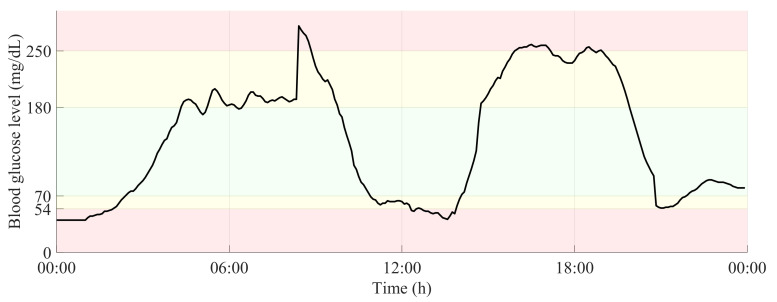
24-h glucose time-series with delimitation of glucose ranges related to normoglycemia and both levels of hypo- and hyperglycemia.

Figure 2 shows a 24-h period of a glucose time-series, in which the glucose ranges related to normoglycemia and both levels of hypo- and hyperglycemia are highlighted.

Following, the vectors corresponding with the composition, obtained from the 24-h time-series. The composition was defined according to Equation (4). First, the distribution of the 288 samples, followed by its equivalent summed to 1440 min (24-h) and last, normalized to unity.

$$x_{samples}=\left(40,40,87,97,24\right)$$

$$x_{minutes}=\left(200,200,435,485,120\right)$$

$$x_{normalized}=\left(0.1389,0.1389,0.3021,0.3368,0.0833\right)$$

The computation of the clr and ilr transformations for these compositions, according to Equations (2) and (3), defined by Equations (5)–(8).

$$\mathrm{clr}\left(x\right)=\left(-0.2304,-0.2304,0.5466,0.6554,-0.7412\right)$$

$$\mathrm{ilr}\left(x\right)=\left(-0.4207,0,-0.4813,-0.9876\right)$$

After the representation in coordinates, an exploratory analysis has been performed. A very useful exploratory tool that allows the discovery of potential clusters of similar compositions and significant statistical relationships between log-ratios of the parts is the clr-biplot [31]. The clr-biplot is an adaptation of the biplot [32] for compositional data.

K-means algorithm [33] was applied to coordinates of 6-h periods at all times to check for different patterns of periods. The algorithm was also applied to the 24-h periods starting at 00:00 and ending at 24:00 to check for different patterns of days. Since the data now is represented through coordinates, the distance between two periods can be easily calculated as the Euclidean distance between two coordinates, meaning that k-means can be directly applied either to the ilr or clr coordinates [34]. The algorithm was tested for several groups considering each time 25 random repetitions of the selection of initial centers. Clustering results can be evaluated using either external or internal validation, in which external information is provided or only the information within the data set is used for clustering validation, respectively. Three different indices that are used for internal validation are the Calinski–Harabasz index [35], Dunn index [36] and Silhouette index [37], those indices have been recently used as validation methods to CoDa clusters in [38,39]. In this work, the choice of k considered, the interpretability of the clinical outcomes of the groups of both 6-h and 24-h and their distribution in the clr-biplot.

Groups of periods with different lengths were analyzed regarding the maximums and minimums of parts and ratios. Additional measures were also obtained to improve the interpretation of the 24-h periods: average blood glucose (BG), BG variation (BGV), Low and High Blood Glucose risk Indexes (LBGI and HBGI) [40], total basal insulin, total bolus insulin, time of pump suspension and total carbohydrate (CHO) ingested.

As stated, k-means algorithm was applied separately for periods of 6-h and 24-h, therefore, the categorization for periods of different duration was done independently. Once the categories of the 24-h periods were obtained, a discriminant analysis method was applied to each patient’s 24-h periods from 00:00 to 24:00. This discriminant analysis considered only 24-h periods from 00:00 to 24:00 and was used to find a discrimination rule to assign any individual 24-h composition (**x**) at different times (00:00, 06:00, 12:00, and 18:00) to a group. The main objective is to make possible that the individual looks at the previous 24-h composition, at different times of the day and determine the group to which that 24-h period belongs.

We considered a linear discriminant (LD) model where the discrimination rule is based on compositional linear functions on **x** [34]. Discriminant analysis was developed by [41] and it is one of the most traditional methods for classification. The discriminant rule is based on probabilities. One composition **x** is classified in a determined group with the largest probability, using the information provided by the training data set. The input features considered for the LD classifier were the ilr coordinates. The discrimination functions used to classify the data were calculated considering the information included in the composition using leave-one-out cross-validation (LOOCV). The accuracy of the method was measured by the percentage of compositions correctly classified in its respective group.

The probability model of transition between a category of the previous 24-h period to a subsequent 6-h period was performed through a retrospective analysis of the data after the proper categorization of the periods. This analysis was performed at different times of the day: 00:00, 06:00, 12:00, and 18:00. The counts of moving from determined category of 24-h period to a category of 6-h period were expressed it in terms of probabilities of transition at different times of the day. A probabilistic model of transition between the category of the past 24-h of glucose to the category of the future 6-h period was obtained.

## 4. Results

This section is organized as follows: first, a general description of the groups obtained for periods of both 24-h and 6-h duration is presented. For periods of both durations, the main characteristics of each group obtained are presented. Then, the results of the LD model used for the categorization of glucose profiles are presented. Finally, a probability model that represents the probabilities of transition from the preceding 24-h period to the following 6-h period is presented.

### 4.1. General Description of the Periods

Both 24-h and 6-h periods of each patient were categorized separately. The periods were characterized in terms of relative time spent by the individuals in specific glucose ranges during 24-h or 6-h, according to the log-ratio approach. Even though groups from different patients may present comparable characteristics regarding the relative interpretation of time spent in different glucose ranges, the results must be interpreted per patient and in a relative sense and not in an absolute way.

#### 4.1.1. 24-H Periods

The 24-h periods were categorized in five different groups, described as V, W, X, Y, and Z:

- V - periods with relatively high amounts of time in level 2 hypoglycemia
- W - periods with relatively high amounts of time in level 1 hypoglycemia
- X - periods with relatively low amounts of time in the ranges related to hypo- and hyperglycemia
- Y - periods with relatively low amounts of time in the ranges related to hypo- and normoglycemia and high amounts of time in the ranges related to hyperglycemia.
- Z - periods with relatively high amounts of time in the ranges related to level 1 hypoglycemia and both levels of hyperglycemia.

Table 6 shows the log-ratios between the center of each group and the overall center for all patients (Table 3). Positive values reflect the relative mean of a part above the overall composition and negative values reflect the relative mean of a part below the overall composition.

The values of Table 6 can be interpreted in terms of time: the values below 54 mg/dL (level 2 hypoglycemia) during periods of type V in patient P5 are approximately 61 ($\exp\left(4.12\right)$) times the average time in this range considering the mean composition. This value decreases to 6% ($\exp\left(-2.70\right)$) when data in group Y for the same patient are considered. Considering the mean values showed in Table 3, during 24-h periods of group V of P5, this patient spends on average approximately 37 min in level 2 hypoglycemic range.

Table 7 shows the summary of glucose and insulin outcomes per group for 24-h periods. The lowest Avg BG is presented for periods of group W for all patients, except for patient P5, in which the lowest Avg BG is presented for periods of type V. The highest Avg BG is presented for periods of group Y for all patients. The greatest BGV is presented in periods of group V for patients P1, P2, and P4. Periods of type V of patients P2, P4, and P5 presented a moderate LBGI (LGBI between 2.5 and 5.0), and periods of type Y of all patients presented the highest HBGI between all groups; however, only for P1, P3, and P6 this index was considered high (HBGI > 9.0). All three groups of patient P5 presented high HBGI. Periods of type V of patients P1, P2, P3, and P5 presented the lowest amount of basal insulin during the 24-h periods, while the periods of type Y of patients P2, P3, and P5 presented the highest amount of bolus insulin. Periods of group W of P2, P3, and P4 presented the highest amounts of CHO ingested.

#### 4.1.2. Categories of 6-h Periods

The 6-h periods were categorized in four different groups, described as A, B, C and D, according to the comparison of the compositional center of each group with the whole center:

- A—periods with relatively high amounts of time in both levels of hypoglycemia and relatively low amounts of time in both levels of hyperglycemia
- B—periods with relatively low amounts of time in both levels of hypoglycemia and level 2 hyperglycemia
- C—periods with relatively high amounts of time in both levels of hyperglycemia
- D—periods with relatively high amounts of time in both levels of hypoglycemia and low amounts of time in level 1 hyperglycemia.

Table 8 shows the compositional center of each group of 6-h periods for all patients. the geometric mean of the amounts of time spent in each glucose range, obtained by adjusting the geometric mean of components to 360 min.

It should be emphasized that times in different glucose ranges showed in Table 8 are geometric means of groups composed by several periods. The 6-h periods of group A of all patients (except P4) are characterized by the fact that individuals during periods of type A spent longer time in both levels of hypoglycemia than during periods of other types. For P4, the average time spent in level 2 hypoglycemia during periods of type D is even higher than in periods of type A.

For all patients that presented 6-h periods of type D, except P5, even though most of the time was spent by the individuals in the target range, a parcel of the time during some of these 6-h periods was spent with BG below 54 mg/dL. Time spent in level 2 hypoglycemia would require immediate action to be taken by the patient.

### 4.2. Linear Discriminant Model

The classification accuracy obtained per patient with the linear discriminant model is showed in Table 9. These models were obtained with the valid 24-h periods starting at 00:00 and ending at 24:00, regardless of the existence of valid 6-h periods in sequence. LOOCV and the respective categories obtained for these periods were considered.

The LD models obtained are suitable for the categorization of glucose profiles, as shown in Table 9, which expresses high accuracy in classification.

### 4.3. Transition between Periods

The transition between the categories of the last 24-h period to the category of the subsequent 6-h period has been analyzed at different times of the day: 00:00, 06:00, 12:00, and 18:00. These transitions were counted and Table 10 shows the probabilities of transition at different times of the day for all patients.

Consider patient P2, at 12:00. The patient analyzes his glucose composition from his previous 24-h period, and verify that this period is categorized as type W. Currently, the probability of the category of the next 6-h period, from 12:00 to 18:00, being of type A is equal to 71.43%. As previously shown in Table 8, during 6-h periods of type A, patient P2 experiences, on average, 28 min of hypoglycemic events of level 1. If the patient knows the category of his previous 24-h period, and it is expected that he will continue with unsatisfactory glycemic control during the subsequent period, he/she may take a corrective action (that is, adjust basal insulin, take some carbohydrates, or correction bolus), to avoid that.

Consider patient P5, at 00:00. The patient analyzes his glucose composition and verify that the last 24-h are categorized as type W. During days of type W, even though P5 presents high Avg BG and BGV (Table 7), these days are also characterized by the fact this subject spends relatively high amounts of time experiencing hypoglycemic events of level 1 (Table 6). At this time, if a day of type W is detected, the probability of the next 6-h period (from 00:00 to 06:00) be of type A is approximately 57%. So if the patient knows that he has a nearly 60% chance of experiencing a hypoglycemic event during sleep time, he might take some carbohydrates before sleeping or even adjust the basal insulin during nighttime.

There are situations, however, in which it is probably better to avoid any special action. For instance, consider patient P4, at 18:00, even though the probability of moving from a day of type Y to a period of type C is the highest: 30.77%, and both types, Y and C, are characterized by high amounts of time in the ranges related to hyperglycemia, the probability of moving to a period related to hypoglycemia is almost as high: 23.08%, i.e., if the individual decides to increase the insulin aiming to avoid undesired hyperglycemia, it must be taken into account that the risk of that action will result in a hypoglycemic event is nearly the same.

## 5. Conclusions

A probabilistic model of transition between categories of glucose data has been presented in this work. This methodology is based on a previously presented methodology for the categorization of glucose profiles using compositional data analysis. In this work, we obtained a linear discriminant model suitable for the categorization of glucose data. A novel approach for the retrospective analysis of transition between categories of subsequent periods of different duration has been presented. The analysis was performed considering a dataset composed of 24-h and 6-h periods of glucose data, at different times of the day: 00:00, 06:00, 12:00, and 18:00. These times were chosen globally for all patients to comprise approximations of daytimes where relevant events occur, such as sleep time, waking up and breakfast, lunch, and dinner.

It is important to highlight that the probability model of transition between categories of 24-h to 6-h periods was retrospectively obtained from the available periods in sequence. Due to the limitation of the dataset, there were cases in which the probabilities values between different transitions were very similar. Additionally, the validation of the probability model has not been presented yet. The dataset used in this study has been acquired for different purposes. It was upon retrospective analysis that the authors considered the dataset, initially, for the categorization of profiles, and later, for the definition of the probability model of transition. The authors are aware that the number of patients involved in the study (six) is too small to represent the whole T1D patient population. However, this work aimed to present the feasibility of an individualized methodology that allows the analysis of transitions between different categories (or conditions) of both days and periods of a single patient. The categorization of glucose daily profiles has been presented earlier [13,14], this work shows that the discrimination of the past 24-h period is accurate and feasible to be performed at different times of the day. That is, in a real-time application, it is possible for the patient to look at his glycemic profile of the last 24-h, and to be able to identify in which category this period reliably fits. Then, the patient can use the transition model to predict the category of the future period and take corrective actions accordingly. The authors foresee that with a more expressive dataset, a probabilistic model of transitions could be validated with more representative results, allowing the application of the methodology into a decision support tool for managing T1D.

The prediction of blood glucose values using regression algorithms, considering glucose trends and other information regarding insulin therapy, has been pointed out by Oviedo et al as the most widely used for glucose management purposes [42]. This work, however, is not intended to present the prediction of blood glucose values or trends. The work follows a new approach for the identification of glycemic patterns based on a new method that considers the relative interpretation of time spent in different glucose ranges, based on CoDa analysis, as has been initially proposed by [13,14]. Followed by that, the main objective is to be able to predict the condition of the patient in the future, allowing the adjustment of therapy parameters according to the prediction, and for that, this work presents a new probability model of transition between categories of periods. Additionally, it is essential to recall that even though the results of groups of periods were presented considering common descriptions between patients, the results must be interpreted individually per patient, which would allow personalized analysis and assessment of patients’ state, prediction of future condition, and individualized adjustment of therapies

It was a first approach for the obtainment of a probabilistic model of transition between categories at different times of the day. In the future, the models can be adjusted, considering flexible periods according to patients’ routine. The approach is suitable for the categorization of glucose profiles and it can work as a new analysis tool. Additionally, it works as a complementary tool for the prediction of different categories of glucose control, which could assist patients to take correction measures ahead of adverse situations.

The methodology proposed could be incorporated into a DSS that would be able to identify patients’ current condition based on the distribution of time spent in different glucose ranges and to provide a probability of transition to another condition in the subsequent period. Therefore, following the definition of patients’ current category of glucose control, the transition model provides the prediction of patient’s condition in the future, which would allow tailored adjustments of therapy parameters according to the expected condition of the patient in the next few hours. In the future, the methodology could be applied for both open-loop and closed-loop systems (i.e., by suggesting ingestion of CHO, adjustments on insulin dosing, or modifications in the controller’s parameters). For example, if the patient expects a high probability that the future few hours will be characterized by the experience of hypoglycemic events, he might be able to decrease his basal insulin levels to try to avoid the hypoglycemic events. Additionally, if the patient expects that the next few hours will be characterized by high glucose variability, he can be less aggressive with his insulin boluses.

Even though the analysis was performed considering a limited dataset obtained from glucose data of six patients, the results could be used in the future. Over time, when additional data are available, it is possible that there could be changes in the number of clusters of each patient, indicating alterations in patients’ behavior and physiology. Following that, the probabilistic model of transitions could be periodically updated during the visits to the physicians. Likewise, the availability of new data could also support the creation of models with a fixed quantity of periods in analysis, where the incorporation of more recent data would imply the disposal of the earliest data. Nevertheless, a more representative model of transitions between periods would be required, including additional analysis of the insulin therapy of each patient and a more extensive dataset. The usage of this information could assist patients to identify evidence-based actions that would be of benefit and to anticipate the occurrence of adverse events. Additionally, it could support physicians to assess patients’ outcomes and tailor their insulin dosing profile.

## Figures and Tables

**Table 1:** Demographic characteristics for the dataset used.

Number of T1D patients * (females)	6 (1)
Age (years)	36.7 ± 8.9
HbA1c (%)	7.9 ± 0.5
BMI (kg/m^2^)	24.6 ± 1.0
Time with T1D (years)	25.2 ± 12.7
Time with pump (years)	4.8 ± 1.7

* All patients who participated in the study were Caucasian.

**Table 2:** Standardized clinical levels of hypo- and hyperglycemia [23,24].

Hyperglycemia		Hypoglycemia	
**Level 1:** Elevated glucose	180 < BG ≤ 250	**Level 1:** Measurable glucose concentration that can alert a person to take action	54 ≤ BG < 70
**Level 2:** Very elevated glucose	BG > 250	**Level 2:** Measurable glucose concentration that needs immediate action	BG < 54

BG values in mg/dL.

**Table 3:** Compositional center of each patient. Analysis of 24-h periods from 00:00 to 24:00. Geometric mean of the amounts of time spent in each glucose range, obtained by adjusting the geometric means of the components to 1440 min (24-h).

	<54	54–70	70–180	180–250	>250
	(min)	(min)	(min)	(min)	(min)
P 1	$3.7\times 10^{-2}$	11.1	962.0	443.0	23.9
P2	$4.9\times 10^{-2}$	7.4	1235.4	189.9	7.3
P3	$1.2\times 10^{-9}$	$5.0\times 10^{-2}$	1170.9	264.9	4.1
P4	$6.3\times 10^{-7}$	15.5	1075.4	310.2	38.9
P5	0.6	5.0	820.4	439.0	175.1
P6	$1.2\times 10^{-5}$	2.1	805.0	568.0	64.9

**Table 4:** Quantity of 24-h periods followed by a 6-h period at each time of analysis available per patient.

Quantity of Valid 24-h Periods		6-h Periods in Sequence according to Time			
		00:00	06:00	12:00	18:00
P1	92	24	22	24	22
P2	122	29	31	31	31
P3	184	47	44	48	45
P4	185	48	47	49	41
P5	132	30	32	35	35
P6	55	15	12	14	14

**Table 5:** Sequential Binary Partition defined for the log-ratio coordinates.

*i*	G.54	G.54.70	G.70.180	G.180.250	G.250
1	+1	+1	−1	−1	−1
2	+1	−1	0	0	0
3	0	0	−1	+1	+1
4	0	0	0	−1	+1

**Table 6:** Comparison between the compositional center of each group with the whole center. Values are expressed per part and represent relative proportion to the mean composition.

		<54	54–70	70–180	180–250	>250
	V	7.32	1.67	−0.21	0.13	1.03
P1	W	−1.77	1.12	0.27	−0.19	−3.05
k = 4	X	−1.92	−2.82	0.00	0.37	−2.98
	Y	−1.69	−0.55	−0.05	−0.05	1.84
	V	7.10	2.51	0.03	−0.16	0.57
P2	W	−1.62	1.78	0.17	−1.07	−3.16
k = 4	X	−3.71	−3.33	0.05	0.36	−2.73
	Y	−2.28	−1.90	−0.16	0.79	2.90
	V	22.02	5.14	0.10	−2.76	0.79
P3	W	0.88	6.50	0.14	−0.13	−1.73
k = 4	X	−5.08	−5.31	0.13	0.19	−2.69
	Y	−1.63	−4.63	−0.27	0.54	3.65
	V	17.65	1.95	−0.33	0.29	0.61
	W	−3.12	1.39	0.18	−0.30	−3.17
P4	X	−6.37	−2.89	0.28	−0.76	−0.06
k = 5	Y	−0.76	−3.21	−0.14	0.65	0.46
	Z	−1.33	1.13	−0.04	0.08	1.38
	V	4.12	3.08	0.08	−0.43	−0.89
P5	W	0.60	2.26	0.18	−0.11	0.46
k = 3	Y	−2.70	−3.14	−0.16	0.31	0.21
	W	0.10	−0.40	0.09	0.30	−3.78
P6	X	−0.19	−2.21	−0.27	0.34	0.89
k = 3	Y	0.20	3.33	0.33	−0.66	1.01

k is the number of groups per each patient. V, W, X, Y, and Z are the groups obtained for the 24-h periods after clustering.

**Table 7:** Summary of clinically relevant outcomes.

	Group	# Days	Avg BG	BGV	LBGI	HBGI	Total	Total	Time Pump	Total
			(mg/dL)	(mg/dL)			Basal	Bolus	Suspension	CHO
							(U)	(U)	(min/Day)	(ex)
	V	5	163.79	64.98	2.35	8.69	20.28	17.20	190.00	13.90
P1	W	6	144.78	43.79	0.86	4.40	20.68	15.17	165.83	13.75
k = 4	X	3	158.77	44.04	0.41	6.25	21.06	15.87	91.67	14.00
	Y	12	173.53	54.45	0.39	9.28	21.28	16.19	74.58	13.88
	V	8	135.44	58.29	2.53	4.69	20.13	22.17	136.88	20.06
P2	W	9	130.25	34.00	0.93	2.53	20.69	20.04	46.11	21.44
k = 4	X	4	158.46	31.85	0.17	5.26	22.93	21.70	0.00	16.50
	Y	12	163.23	56.88	0.51	7.70	21.53	23.59	31.67	19.17
	V	3	150.28	40.52	0.72	5.53	18.50	21.27	98.33	12.17
P3	W	18	140.40	40.50	0.86	4.12	19.46	20.65	67.50	14.43
k = 4	X	11	151.33	36.34	0.23	4.77	19.91	20.22	49.55	13.23
	Y	16	181.44	49.86	0.10	9.97	19.95	24.33	30.63	13.71
	V	6	148.75	63.04	2.89	7.20	20.55	27.35	30.00	21.43
P4	W	10	133.25	39.94	1.20	3.19	20.97	26.63	3.50	25.62
k = 5	X	7	145.57	45.71	0.24	4.36	20.78	25.90	55.71	22.61
	Y	8	170.07	50.95	0.30	8.13	20.17	24.58	0.00	19.15
	Z	18	161.58	63.81	0.83	7.86	20.00	27.54	14.72	22.06
P5	V	9	161.35	70.17	2.68	9.26	21.04	29.94	92.78	9.67
k = 3	W	10	179.46	76.95	0.83	11.33	21.97	37.21	7.00	13.45
	Y	16	199.67	60.32	0.14	13.64	23.51	43.58	13.13	14.19
P6	W	3	173.71	36.35	0.19	7.85	24.08	23.73	0.00	18.50
k = 3	X	7	192.71	44.94	0.06	11.69	24.04	23.21	0.00	15.79
	Y	5	158.67	60.03	1.22	7.90	23.53	19.80	37.00	15.70

k is the number of groups per each patient. V, W, X, Y, and Z are the groups obtained for the 24-h periods after clustering.

**Table 8:** Compositional center of each group of 6-h periods for each patient. Geometric mean of the amounts of time spent in each glucose range, obtained by adjusting the geometric mean of components to 360 min.

		Avg BG	BGV	<54	54–70	70–180	180–250	>250
		(mg/dL)	(mg/dL)	(min)	(min)	(min)	(min)	(min)
	A	125.75	34.29	0.44	7.62	347.69	4.13	0.13
P1	B	165.77	31.48	$1.1\times 10^{-3}$	0.01	224.39	135.58	0.02
k = 3	C	216.42	45.36	0.02	0.05	25.64	227.31	106.99
	A	116.65	34.85	1.06	28.81	326.82	3.26	0.05
P2	B	156.37	33.36	0.01	0.02	274.79	85.11	0.07
k = 4	C	203.59	47.47	0.03	0.07	63.90	204.32	91.67
	D	127.55	24.97	0.37	0.88	357.22	1.00	0.53
	A	122.05	23.07	0.41	1.96	356.30	1.12	0.21
P3	B	166.04	28.84	$1.5\times 10^{-3}$	$4.8\times 10^{-3}$	237.64	122.32	0.04
k = 3	C	212.62	41.85	0.04	0.07	32.15	225.66	102.07
	A	115.68	34.30	0.24	36.71	319.26	3.74	0.05
P4	B	156.93	30.19	$1.7\times 10^{-3}$	0.01	263.91	96.01	0.07
k = 4	C	204.07	53.86	0.01	0.01	112.09	155.71	92.18
	D	129.46	32.97	0.46	1.78	354.25	2.34	1.17
	A	121.33	37.48	1.80	15.25	339.33	3.38	0.23
P5	B	163.03	31.24	$6.5\times 10^{-4}$	$2.8\times 10^{-3}$	241.65	118.26	0.09
k = 4	C	202.56	55.18	$1.3\times 10^{-3}$	$4.6\times 10^{-3}$	148.24	140.38	71.38
	D	279.50	36.66	0.01	0.02	0.24	44.60	315.13
	A	111.01	33.51	0.22	50.69	306.40	2.65	0.04
P6	B	163.05	29.33	$2.0\times 10^{-3}$	0.01	247.95	111.98	0.06
k = 4	C	211.95	39.92	0.02	0.03	24.54	252.86	82.56
	D	131.34	29.15	0.36	1.75	354.77	2.15	0.97

k is the number of groups per each patient. A, B, C, and D are the groups obtained for the 6-h periods after clustering.

**Table 9:** Accuracy of the linear discriminant models with LOOCV.

	Accuracy (%)
P1	100
P2	93.93
P3	97.92
P4	93.87
P5	97.14
P6	86.67
Average	94.92

**Table 10:** Probabilitiesof transition from the preceding 24-h period to the following 6-h period. Analysis at 00:00, 06:00, 12:00 and 18:00.

	To	At 00:00				At 06:00				At 12:00				At 18:00			
From		A	B	C	D	A	B	C	D	A	B	C	D	A	B	C	D
	V	40.00	40.00	20.00		20.00	60.00	20.00		50.00	33.33	16.67		50.00	25.00	25.00	
P1	W	60.00	40.00	0.00		0.00	66.67	33.33		16.67	66.67	16.67		50.00	12.50	37.50	
k = 4	X	33.33	66.67	0.00		100.00	0.00	0.00		0.00	0.00	100.00		0.00	100.00	0.00	
	Y	36.36	27.27	36.36		40.00	40.00	20.00		36.36	45.45	18.18		55.56	11.11	33.33	
	V	0.00	20.00	40.00	40.00	60.00	0.00	0.00	40.00	50.00	0.00	16.67	33.33	50.00	0.00	25.00	25.00
P2	W	11.11	33.33	33.33	22.22	11.11	33.33	33.33	22.22	71.43	28.57	0.00	0.00	50.00	20.00	0.00	30.00
k = 4	X	0.00	50.00	0.00	50.00	20.00	60.00	0.00	20.00	20.00	40.00	40.00	0.00	0.00	33.33	0.00	66.67
	Y	27.27	36.36	9.09	27.27	41.67	25.00	16.67	16.67	23.08	38.46	23.08	15.38	10.00	40.00	20.00	30.00
	V	33.33	0.00	66.67		100.00	0.00	0.00		0.00	0.00	100.00		0.00	0.00	100.00	
P3	W	50.00	50.00	0.00		47.06	47.06	5.88		22.73	59.09	18.18		66.67	28.57	4.76	
k = 4	X	44.44	22.22	33.33		72.73	27.27	0.00		16.67	33.33	50.00		50.00	16.67	33.33	
	Y	41.18	35.29	23.53		13.33	66.67	20.00		15.38	61.54	23.08		35.29	29.41	35.29	
	V	33.33	16.67	16.67	33.33	0.00	40.00	20.00	40.00	28.57	28.57	42.86	0.00	50.00	0.00	50.00	0.00
	W	20.00	30.00	30.00	20.00	9.09	27.27	36.36	27.27	42.86	57.14	0.00	0.00	50.00	20.00	10.00	20.00
P4	X	20.00	40.00	0.00	40.00	0.00	33.33	0.00	66.67	40.00	0.00	20.00	40.00	33.33	0.00	66.67	0.00
k = 5	Y	25.00	50.00	12.50	12.50	27.27	27.27	18.18	27.27	7.69	30.77	46.15	15.38	18.18	36.36	18.18	27.27
	Z	21.05	47.37	15.79	15.79	41.18	29.41	11.76	17.65	5.88	41.18	41.18	11.76	23.08	23.08	30.77	23.08
	V	37.50	25.00	25.00	12.50	33.33	16.67	33.33	16.67	33.33	25.00	33.33	8.33	57.14	7.14	14.29	21.43
P5	W	57.14	28.57	14.29	0.00	16.67	33.33	33.33	16.67	22.22	33.33	33.33	11.11	0.00	37.50	62.50	0.00
k = 3	Y	20.00	20.00	33.33	26.67	21.43	14.29	50.00	14.29	35.71	28.57	28.57	7.14	7.69	30.77	38.46	23.08
	W	33.33	33.33	0.00	33.33	50.00	0.00	0.00	50.00	33.33	0.00	66.67	0.00	0.00	50.00	0.00	50.00
P6	X	0.00	28.57	57.14	14.29	0.00	71.43	28.57	0.00	14.29	71.43	14.29	0.00	14.29	28.57	57.14	0.00
k = 3	Y	0.00	40.00	20.00	40.00	0.00	33.33	66.67	0.00	25.00	0.00	25.00	50.00	20.00	40.00	20.00	20.00

k is the number of groups per each patient. A, B, C, and D are the groups obtained for the 6-h periods after clustering. V, W, X, Y, and Z are the groups obtained for the 24-h periods after clustering.

## Data Availability

Restrictions apply to the availability of these data. Data was obtained from the Spanish Consortium on Artificial Pancreas and Diabetes Technology and are available on request from the corresponding author with the permission of the Spanish Consortium on Artificial Pancreas and Diabetes Technology.

## Abbreviations

The following abbreviations are used in this manuscript:

T1D	Type 1 diabetes
BG	Blood glucose
MDI	Multiple daily injections
CSII	Continuous subcutaneous insulin infusion
CGM	Continuous glucose monitoring
CoDa	Compositional Data
DSS	Decision support system
clr	Centered log-ratio
ilr	Isometric log-ratio
SBP	Sequential binary partition
BGV	Blood glucose variation
LBGI	Low blood glucose risk index
HBGI	High blood glucose risk index
CHO	Carbohydrate
LD	Linear discriminant
LOOCV	Leave-one-out cross-validation
